# Novel AAV843 Vector-Mediated Gene Replacement Therapy Rescues Primary Hyperoxaluria Type I in Mice

**DOI:** 10.3390/cells15070629

**Published:** 2026-03-31

**Authors:** Jingjia Zhang, Ye Yin, Baowei Ji, Manqing Sun, Yuanyan Jiang, Xiaohui Wu, Xiao Xiao, Qian Shen, Xia Wu, Hong Xu

**Affiliations:** 1 Department of Nephrology, Children’s Hospital of Fudan University, National Children’s Medical Center, Shanghai 201102, China; 25111240024@m.fudan.edu.cn (J.Z.);; 2 Shanghai Kidney Development & Pediatric Kidney Disease Research Center, Shanghai 201100, China; 3 Institute of Developmental Biology and Molecular Medicine, Fudan University, Shanghai 200438, China; 4 School of Biotechnology, East China University of Science and Technology, Shanghai 200237, China; 5 School of Pharmacy, East China University of Science and Technology, Shanghai 200237, China; 6 National Key Laboratory of Kidney Diseases, Beijing 100853, China

**Keywords:** primary hyperoxaluria type I, gene replacement therapy, AAV843, kidney injury

## Abstract

Primary hyperoxaluria type 1 (PH1) is a rare autosomal recessive disorder resulting from mutations in the alanine–glyoxylate aminotransferase (*AGXT*) gene, leading to excessive systemic deposition of calcium oxalate. This condition is an important cause of end-stage renal disease in children and poses a serious threat to patient survival. Current therapeutic approaches are limited primarily to conservative management and organ transplantation, underscoring the need for more effective treatments. Gene replacement therapy represents a promising alternative strategy. In this study, *Agxt*^−^/^−^ mice lacking exons 3–8 were characterized, confirming the complete loss of hepatic alanine–glyoxylate aminotransferase (AGT) expression and the presence of early-onset hyperoxaluria. Exposure to glyoxylic acid induced pronounced renal calcium oxalate crystal deposition, establishing a robust and previously unreported induction approach in gene-edited PH1 mouse models. Using this platform, human *AGXT* cDNA was delivered via the liver-tropic, low-immunogenic AAV843 vector, which has been employed in clinical trials for hemophilia. Gene therapy resulted in normalization of urinary oxalate levels, restoration of hepatic AGT expression, and significant attenuation of renal injury and nephrocalcinosis. These therapeutic effects were accompanied by significant suppression of key mediators involved in renal inflammation, necroptosis, and fibrosis. Moreover, transgene expression was highly specific to the liver and was not associated with hepatotoxicity. These results demonstrate that AAV843-mediated AGXT gene replacement is a safe and effective approach that achieves phenotypic correction in a murine model of PH1, warranting further evaluation in preclinical studies.

## 1. Introduction

Primary hyperoxaluria type I (PH1) is a rare autosomal recessive metabolic disorder caused by mutations in the alanine–glyoxylate aminotransferase gene (*AGXT*), which encodes the hepatic enzyme alanine–glyoxylate aminotransferase (AGT) [1]. Under physiological conditions, AGT catalyzes the conversion of glyoxylate to glycine. Loss of AGT activity results in the accumulation of glyoxylate in hepatocytes, which is subsequently oxidized to oxalate by lactate dehydrogenase (LDH). Excess oxalate is primarily eliminated through the kidneys and readily binds calcium to form insoluble calcium oxalate crystals. Progressive crystal deposition causes renal inflammation and injury, ultimately leading to end-stage renal disease, making PH1 a major contributor to pediatric kidney failure [2,3]. Current management strategies, including urinary alkalinization, high fluid intake, and pyridoxine supplementation, do not adequately correct the underlying enzymatic defect. Although combined liver–kidney transplantation remains the most effective therapeutic option, it requires organs from a single donor and is associated with significant surgical risk and long-term immunological complications [4,5,6,7].

Recent developments in gene-based therapies have introduced alternative approaches for PH1 treatment. RNA interference (RNAi) therapeutics, such as lumasiran and nedosiran, reduce hepatic oxalate production by targeting glycolate oxidase (GO) and LDH, respectively, lowering urinary oxalate excretion [8,9]. Despite their efficacy, these agents require lifelong, repeated dosing due to the inherent instability of RNA molecules and impose a substantial financial burden [10]. Genome-editing strategies, such as single-base editing, have demonstrated the ability to correct specific *Agxt* point mutations and reduce oxalate accumulation [11]. However, its applicability is limited to defined mutation types, and unresolved concerns remain regarding off-target effects and unintended genomic alterations [4,12,13,14].

In contrast, *AGXT* cDNA delivered by non-integrating vector gene replacement therapy offers significant clinical potential due to its non-integration, sustained expression, and precise hepatic targeting. Previous studies employing adeno-associated virus (AAV) serotypes 8 and 5, or helper-dependent adenoviral vectors have shown reductions in urinary oxalate levels in murine models [15,16]. However, these investigations did not comprehensively assess renal injury or elucidate underlying pathogenic mechanisms, and pre-existing or therapy-induced neutralizing antibodies (NAbs) have hindered broader clinical translation [17,18].

The AAV843 vector used in this study is an engineered adeno-associated virus characterized by its low immunogenicity. In a clinical investigation involving 154 participants, AAV843 elicited minimal neutralizing antibody induction, with titers substantially lower than those observed with the naturally occurring serotype AAV8 [19]. This vector has also been applied in clinical trials for hemophilia and has received regulatory approval from the National Medical Products Administration (NMPA) [20,21].

In this study, we aimed to characterize the phenotypic outcomes of *Agxt* exon 3–8 deletion in a newly established murine model of PH1, optimize an induction strategy for renal calcium oxalate deposition, and evaluate the therapeutic efficacy and safety of AAV843-mediated *AGXT* gene replacement for phenotypic rescue.

## 2. Materials and Methods

### 2.1. Plasmid Construction and AAV843 Production

Plasmid construction was performed by enzymatic digestion of the vector backbone, amplification of the *AGXT* insert, and subsequent ligation of the fragment into the vector, followed by bacteriophage-based PCR screening for clone identification. The primer sequences used were as follows: AAV-hAGXT-F, TGAATTCCCCGGGGATCCAGTGCAGCCCCAGGT; and AAV-hAGXT-R, GATGACGACAAATGATCTAGAGAGTCGACCTG. The AAV843 capsid was selected from a shuffled AAV capsid gene library as previously reported and was obtained from Shanghai Xinzhi BioMed Co., Ltd. (Shanghai, China) [20,22,23]. Recombinant AAV production, purification, and titration were carried out according to established procedures [24]. Briefly, HEK293T cells were co-transfected with the T02 plasmid encoding the AAV843 capsid, the P11 helper plasmid, and the hAAT-AGXT cDNA expression construct. Cells were collected 72 h after transfection and treated with Benzonase allosteric nuclease (HaiGene, China; C2001) for 3 h, followed by cell disruption using an ultrasonic homogenizer (SCIENTZ-IID, China). Polyethylene glycol 8000 (PEG8000; Sigma, USA; P5413) was then added to a final concentration of 10%, and the suspension was incubated overnight at 2–8 °C. Viral particles were precipitated by centrifugation at 10,000 rpm for 45 min and further purified by iodixanol density gradient ultracentrifugation for 4 h. Recombinant AAV vectors were collected from the 40–60% iodixanol interface using a syringe. This purification step was repeated twice, after which the samples were buffer-exchanged into phosphate-buffered saline (PBS) using 100 kDa molecular weight cutoff ultrafiltration devices to obtain the final AAV preparations. Viral genome titers were determined by droplet digital PCR, while vector purity was assessed by silver staining. Analytical ultracentrifugation (AUC) was performed to quantify the proportion of empty capsids.

### 2.2. Animals

All animal procedures were conducted in accordance with institutional guidelines and were approved by the Ethics Committee of Children’s Hospital of Fudan University (Approval No. 2025-EKYY-155). *Agxt* exon 3–8 heterozygous knockout mice (*Agxt*^+^/^−^) on a C57BL/6J background were obtained from GemPharmatech (China). Animals were maintained under standardized housing conditions with a 12 h light/12 h dark cycle, controlled ambient temperature, and free access to standard laboratory chow and water. Renal stone formation was induced in 12-week-old male *Agxt*^−^/^−^ mice and wild-type (WT) littermates using two methods. In the first model, mice received 1% ethylene glycol (EG) in drinking water for two weeks, followed by 1.4% EG for an additional two weeks. In the second model, glyoxylic acid (GA) was administered via intraperitoneal injection once daily for four consecutive days. Mice were randomly assigned to four groups (*n* = 5 per group): *Agxt*^−^/^−^ *+* EG, WT + EG, *Agxt*^−^/^−^ *+* GA, and WT + GA. Animals were subsequently euthanized, and blood and tissue samples were collected for analysis. Based on the outcomes of these modeling experiments, an additional cohort of 12-week-old male *Agxt*^−^/^−^ mice was randomly allocated to treatment and untreated control groups, with WT mice serving as a reference control (*n* = 5 per group). The treatment group received a single tail vein injection of AAV843-hAAT-AGXT, whereas the untreated group received an equivalent dose of the AAV843 empty vector. WT mice received an equivalent volume of physiological saline. All injections were performed at a dose of 5 × 10^12^ vg/kg. Before vector administration, mice were housed individually in metabolic cages for 24 h to collect baseline urine samples. Urine collection was subsequently repeated for 24 h at 1, 2, 4, 8, and 12 weeks post-injection. To induce nephrolithiasis, mice were injected intraperitoneally with glyoxylic acid at 85 mg/kg once daily for 4 consecutive days. They were euthanized three days later for blood and tissue collection. Throughout the experimental period, animals were monitored daily for general health, behavior, and signs of distress. Randomization was performed using Excel’s RAND function. Humane endpoint criteria were predefined and included severe or unrelieved distress, inability to access food or water, or significant impairment of mobility. Any animal meeting these criteria was humanely euthanized immediately to minimize suffering.

### 2.3. Genotyping

*Agxt*^+^/^−^ male and female mice were bred at a ratio of 1:2. Genotyping of the resulting offspring was performed using the AdeptTect Mouse Tissue Fast PCR Kit (Accurate Biotechnology, China, AG12310) in accordance with the manufacturer’s protocol. The primer sequences used for genotyping are provided in Supplementary Table S1.

### 2.4. Western Blotting

Cells and liver tissues were lysed in RIPA buffer (Beyotime, China; P0013B) supplemented with phosphatase inhibitor (Beyotime, P1045) and protease inhibitor (Beyotime, China, P1005). After centrifugation, proteins in the clarified supernatant were resolved by SDS–PAGE and subsequently transferred onto PVDF membranes (Merck Millipore, USA; ISEQ00010). The membranes were blocked with 5% skim milk and incubated overnight at 4 °C with primary antibodies against AGT (Novus Biologicals, USA, NBP1-89200; 1:1500) and GAPDH (Servicebio, China, ZB15004-HRP-100; 1:2500). After three washes with TBST, the membranes were incubated with the appropriate secondary antibody (Cell Signaling Technology, USA, 7074; 1:2000) for 1 h at room temperature. Protein signals were detected with a chemiluminescent substrate, and band intensities were quantified using ImageJ v1.8.0.345.

### 2.5. Real-Time Fluorescence Quantitative PCR (qPCR)

Total RNA was extracted from liver tissues and cultured cells using TRIzol reagent (Invitrogen; USA, 15596018). The cDNA was generated from the isolated RNA with a reverse transcription premix kit (Accurate Biotechnology; China, AG11728) in strict accordance with the manufacturer’s instructions. qPCR amplification was subsequently performed using a premix qPCR kit (Accurate Biotechnology; China, AG11718) on a Roche LightCycler 480II system. The amplification protocol included an initial denaturation step at 95 °C for 30 s, followed by 40 cycles of denaturation at 95 °C for 5 s and annealing/extension at 60 °C for 30 s, with real-time fluorescence detection throughout the reaction. The primer sequences are provided in Supplementary Table S2.

### 2.6. Hematoxylin-Eosin Staining (H&E Staining)

Freshly harvested mouse liver and kidney tissues were fixed in 4% paraformaldehyde, paraffin-embedded, and sectioned. Following deparaffinization, tissue sections were stained with hematoxylin and eosin, mounted, and examined microscopically for histopathological assessment. Renal pathology was evaluated using a semi-quantitative scoring system applied to hematoxylin and eosin-stained sections across four predefined domains: (i) glomerular vascular lesions (0–3 points), graded according to the severity of structural abnormalities, necrosis, and inflammatory cell infiltration; (ii) glomerular atrophy and necrotic crescent formation (0–3 points), based on the extent of glomerular volume reduction and the presence of necrotic crescents; (iii) tubular epithelial injury (0–3 points), assessing epithelial degeneration, necrosis, and disruption of tubular architecture; and (iv) interstitial pathology (0–4 points), scored according to increasing degrees of fibrosis and inflammatory infiltration. Higher composite scores reflected greater histological damage, with the total renal pathology score calculated as the sum of all individual domain scores.

### 2.7. Immunohistochemistry

Fresh mouse liver tissues were fixed overnight in 4% paraformaldehyde, followed by dehydration, paraffin embedding, sectioning, and deparaffinization. Antigen retrieval was performed by heat-induced epitope retrieval in citrate buffer (pH 6.0) at 100 °C for 15 min. After blocking with 3% bovine serum albumin (BSA; Servicebio, China, GC305010) for 30 min, tissue sections were incubated overnight at 4 °C with a primary antibody against AGT (Novus Biologicals; USA, NBP1-89200; 1:100). Sections were then incubated with the corresponding secondary antibody (Cell Signaling Technology; USA, 7074; 1:1000) for 50 min at room temperature. Nuclei were counterstained with DAPI, and the sections were subsequently dehydrated and mounted for imaging.

### 2.8. Data Analysis

Statistical analyses were performed using GraphPad Prism version 9.0. Data are expressed as the mean ± standard error of the mean. Normality of data distribution was assessed using the Shapiro–Wilk test. For comparisons between two groups, an unpaired Student’s *t*-test was applied when the data met the assumptions of normality; otherwise, the Mann–Whitney U test was used. For multiple-group comparisons, normally distributed data were analyzed using one-way analysis of variance (ANOVA), followed by Tukey’s multiple comparisons test for pairwise comparisons among all groups. Non-normally distributed data were analyzed using the Kruskal–Wallis test, followed by Dunn’s multiple comparisons test. A *p*-value < 0.05 was considered statistically significant.

## 3. Results

### 3.1. Construction and In Vitro Validation of the hAAT-AGXT Plasmid

**Figure 1:**
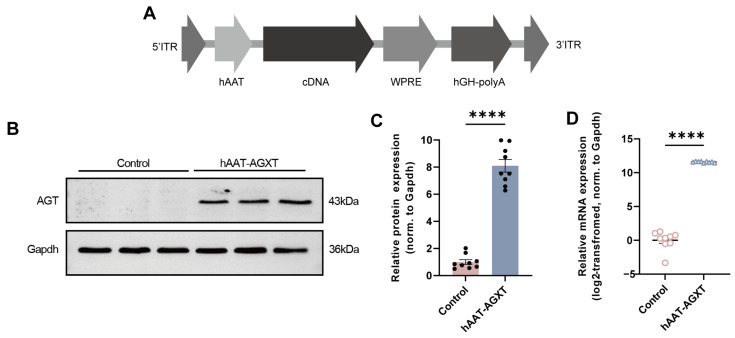
Construction and verification of *AGXT* adeno-associated virus plasmid overexpression. (**A**) Diagram showing the structure of the *AGXT* adeno-associated virus plasmid. The gene expression cassette included inverted terminal repeats (5′ ITR and 3′ ITR), the human α1 antitrypsin (hAAT) promoter, the 5′ untranslated region (UTR), h*AGXT* cDNA, 3 × flag sequences, the woodchuck hepatitis virus post-transcriptional regulatory element (WPRE), and the 3′ human growth hormone polyadenylation signal (hGH-polyA). The plasmid is designated as hAAT-AGXT. (**B**,**C**) Western blots showing AGT protein contents in hAAT-AGXT-transfected cells versus control cells; *n* = 9 per group; Mann–Whitney U test. (**D**) Relative *AGXT* mRNA expression levels in hAAT-AGXT-transfected cells compared to control cells; Unpaired Student’s *t*-test. Data are presented as mean ± standard error, with *n* = 9. **** *p* < 0.0001.

An AAV expression plasmid was generated in which human *AGXT* cDNA was placed under the control of the liver-specific hAAT promoter, yielding the construct termed hAAT-AGXT (Figure 1A). Hepa1–6 cells were transfected with the hAAT-AGXT plasmid. In comparison, cells exposed to the transfection reagent alone served as negative controls. Human *AGXT* expression was subsequently evaluated at both the mRNA and protein levels using qPCR and Western blotting, respectively. Consistent with the construct design, hAAT-AGXT transfection resulted in a significant increase in *AGXT* transcript abundance and AGT protein expression. In comparison, control cells displayed minimal or undetectable *AGXT* expression (Figure 1B–D).

### 3.2. Loss of Hepatic AGT and Elevated Urinary Oxalate Excretion in Agxt^−^/^−^ Mice

**Figure 2:**
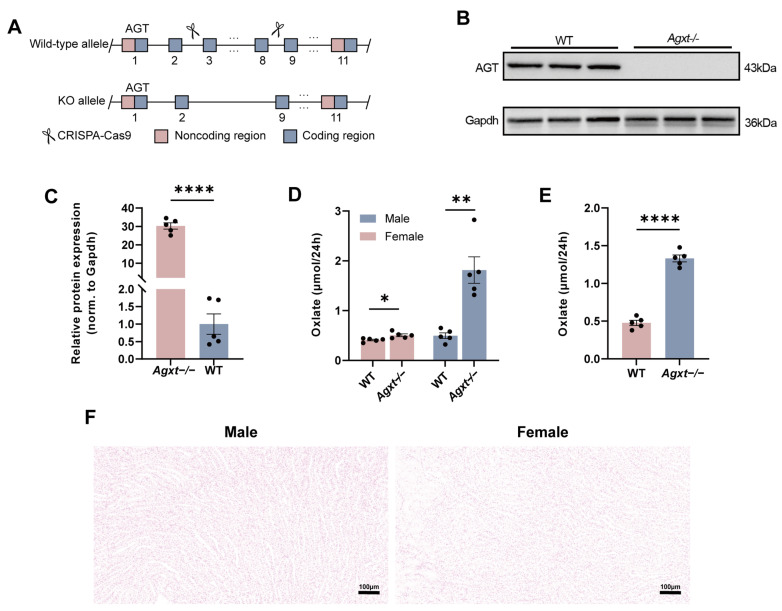
Analysis of hepatic AGT protein contents and urinary oxalate levels in WT and *Agxt*^−^/^−^ mice. (**A**) Schematic diagram of the knockout strategy in *Agxt*^−^/^−^ mice. (**B**,**C**) Western blotting showing AGT levels in liver tissue from 12-week-old WT and *Agxt*^−^/^−^ mice, with quantification; *n* = 5 per group; Mann–Whitney U test. (**D**) Comparison of 24 h urinary oxalic acid levels between 12-week-old WT and *Agxt*^−^/^−^ mice; *n* = 5 per group. (**E**) Comparison of 24 h urinary oxalic acid levels in 4-week-old male *Agxt*^−^/^−^ and WT mice, *n* = 5 per group. (**F**) Kidney Pizzolato staining for the detection of kidney stones in 12-week-old male and female *Agxt*^−^/^−^ mice; *n* = 5 per group. Unpaired Student’s *t*-test (**D**,**E**). Data are presented as mean ± SEM, * *p* < 0.05, ** *p* < 0.01, **** *p* < 0.0001, scale bar = 100 μm.

Previous PH1 mouse models have predominantly relied on the deletion of *Agxt* exons 4–8. In comparison, the *Agxt*^−^/^−^ mice used in this study were generated on a pure C57BL/6 background with exons 3–8 of the *Agxt* gene disrupted using CRISPR–Cas9 technology, encompassing a broader deletion region (Figure 2A) [15,16,25]. Western blot analysis of liver tissues from 12-week-old WT and *Agxt*^−^/^−^ mice demonstrated an almost complete absence of hepatic AGT protein in *Agxt*^−^/^−^ animals, confirming effective gene knockout (Figure 2B,C). At 12 weeks of age, Pizzolato staining of kidney sections from *Agxt*^−^/^−^ mice (*n* = 5 per sex) showed no detectable calcium oxalate crystal deposition (Figure 2F). Despite the lack of overt renal crystal accumulation, urinary oxalate levels were significantly increased in *Agxt*^−^/^−^ mice of both sexes compared with WT controls (Figure 2D). Male *Agxt*^−^/^−^ mice showed substantially higher oxalate excretion than female WT mice (Figure 2D), indicating a sex-dependent difference in disease severity [26,27]. Moreover, this hyperoxaluric phenotype was already evident in male *Agxt*^−^/^−^ mice at 4 weeks of age (Figure 2E), demonstrating the early onset of metabolic dysfunction characteristic of PH1 in this model.

### 3.3. GA Exposure Induced Renal Calcium Oxalate Deposition and Injury in Agxt^−^/^−^ Mice

**Figure 3:**
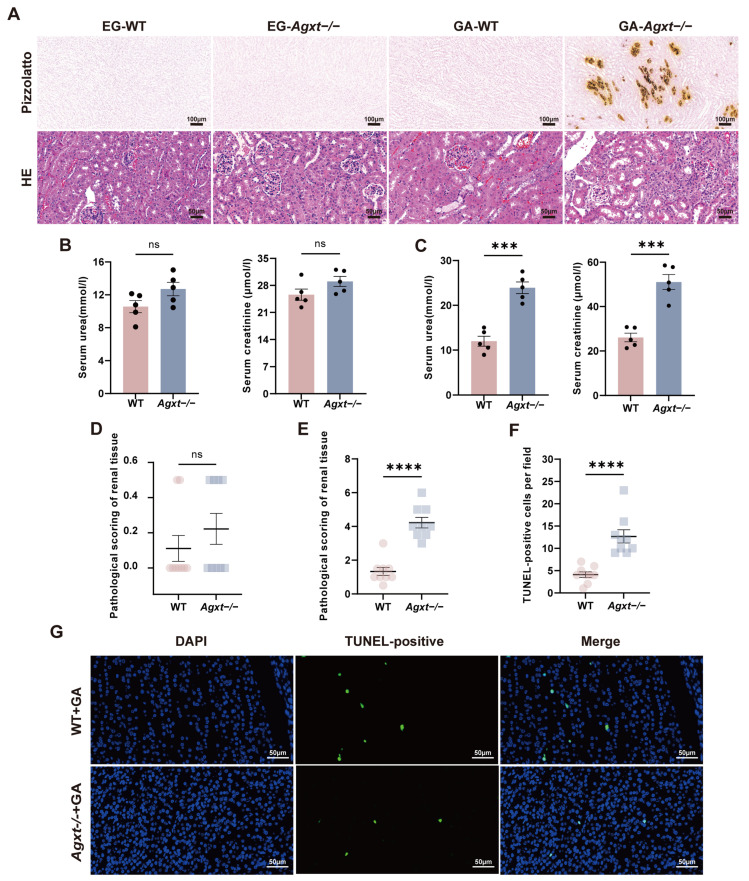
Kidney stone phenotypes induced by EG and GA in male *Agxt*^−^/^−^ mice. (**A**) Representative images of kidney Pizzolato (scale bar = 100 μm) and hematoxylin-eosin staining (scale bar = 50 μm) in 12-week-old male WT and *Agxt*^−^/^−^ mice following EG and GA exposure. Each group included five mice. (**B**,**C**) Comparison of blood urea and creatinine levels in male WT and *Agxt*^−^/^−^ mice 12 weeks after EG and GA exposure, respectively, with *n* = 5 per group; unpaired Student’s *t*-test. (**D**) Semi-quantitative pathological scoring of H&E staining renal sections from WT and *Agxt*^−^/^−^ mice after EG exposure. For quantitative analysis, three samples were randomly selected from each group, with three fields of view randomly chosen for each sample. Mann–Whitney U test. (**E**) Semi-quantitative pathological scoring of HE-stained renal sections from WT and *Agxt*^−^/^−^ mice after GA exposure. For quantitative analyses, three samples were randomly selected from each group, with three fields of view randomly chosen for each sample. Mann–Whitney U test. (**F**,**G**) Representative images of TUNEL staining and enumeration of TUNEL-positive cells in male WT and *Agxt*^−^/^−^ mice 12 weeks after GA exposure. Three samples were randomly selected from each group, with three fields of view randomly chosen for each sample (*n* = 5 per group), and a scale bar of 50 μm. Mann–Whitney U test. Data are shown as mean ± standard error; *** *p* < 0.001, **** *p* < 0.0001. ns, non-significant.

Based on previously reported protocols, we first attempted to induce nephrolithiasis by administering 1% EG in the drinking water for two weeks, followed by 1.4% EG for an additional two weeks [28,29]. Using this approach, only mild renal calcium deposition was observed in a single male *Agxt*^−^/^−^ mouse, and H&E staining revealed no overt renal histopathological damage (Figure 3A,D). Serum urea and creatinine concentrations did not differ significantly among the experimental groups (Figure 3B). Given the limited efficacy of the EG protocol, we next evaluated a GA–based induction strategy, exploiting its role as a metabolic precursor of oxalate [30,31]. Under this regimen, all male *Agxt*^−^/^−^ mice developed pronounced renal calcium deposition. In three animals, crystal accumulation was predominantly localized to the corticomedullary junction, whereas the remaining two demonstrated more diffuse deposition throughout both the cortex and medulla. In comparison, only two of five male WT mice displayed minimal calcium deposits (Figure 3A). Histopathological scoring confirmed significantly greater renal injury in *Agxt*^−^/^−^ mice compared with WT controls (Figure 3E), which was further supported by elevated serum urea and creatinine levels (Figure 3C). Terminal deoxynucleotidyl transferase dUTP nick end labeling (TUNEL) staining revealed a significant increase in apoptotic cells in the kidneys of *Agxt*^−^/^−^ mice (Figure 3F,G). These findings demonstrate that GA administration induces renal calcium deposition, tissue injury, and apoptosis in *Agxt*^−^/^−^ mice more effectively than the conventional EG-based protocol.

**Figure 4:**
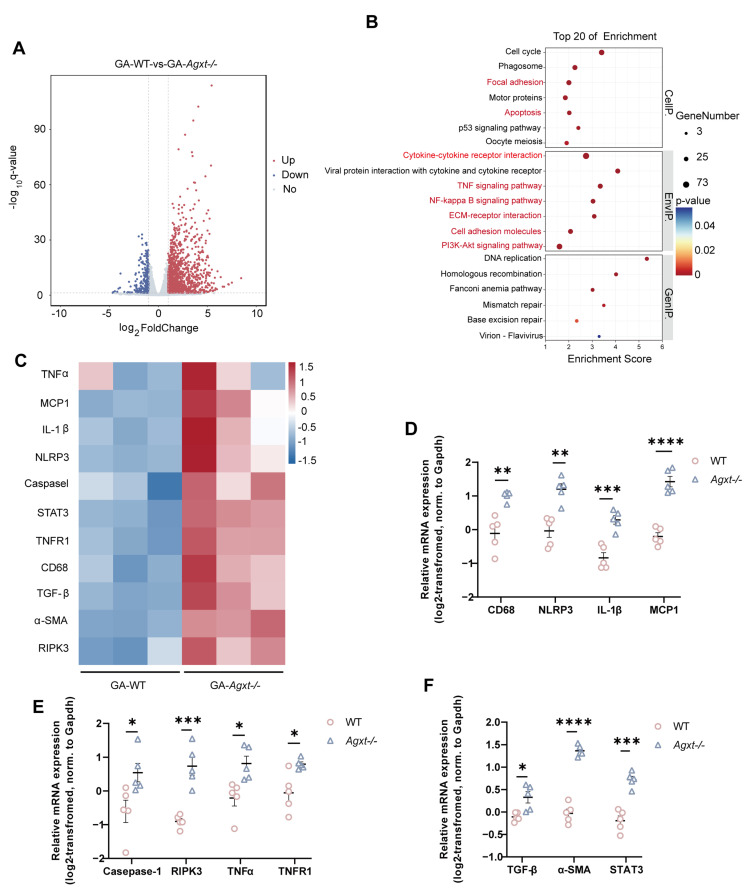
Gene up-regulation associated with kidney injury in *Agxt*^−^/^−^ mice compared to WT mice post-GA induction. (**A**) Volcano plots depicting significantly up-regulated (red) and down-regulated (blue) genes in the kidneys of *Agxt*^−^/^−^ and WT mice following GA induction. (**B**) Differential pathway enrichment analysis in the kidneys of *Agxt*^−^/^−^ and WT mice post-GA induction. (**C**) RNA-seq heatmap showing significantly altered gene expression patterns associated with kidney injury in *Agxt*^−^/^−^ and WT mice after GA induction. (**D**) Expression levels of the factors related to inflammation in WT and *Agxt*^−^/^−^ mice after GA exposure. (**E**) Expression levels of renal factors associated with necrosis in WT and *Agxt*^−^/^−^ mice after GA exposure. (**F**) Expression levels of the factors related to fibrosis in WT and *Agxt*^−^/^−^ mice after GA exposure. *n* = 5 per group. Data are shown as mean ± standard error. Unpaired Student’s *t*-test, * *p* < 0.05, ** *p* < 0.01, *** *p* < 0.001, **** *p* < 0.0001.

Whole-transcriptome RNA sequencing (RNA-seq) was performed on kidney tissues from GA–treated *Agxt*^−^/^−^ and WT mice. Comparative analysis identified 1302 genes that were upregulated and 284 genes that were downregulated in *Agxt*^−^/^−^ mice relative to WT controls (Figure 4A). The Kyoto Encyclopedia of Genes and Genomes (KEGG)pathway enrichment analysis revealed that these differentially expressed genes were significantly associated with multiple signaling pathways, including cytokine–cytokine receptor interaction, extracellular matrix (ECM)–receptor interaction, tumor necrosis factor alpha (TNF-α) signaling, nuclear factor kappa-B (NF-κB) signaling, phosphatidylinositol 3-kinase–protein kinase B (PI3K–Akt) signaling, and focal adhesion pathways (Figure 4B). Genes linked to renal injury were prominently upregulated (Figure 4C), and 12 representative genes were selected for further validation. Quantitative analysis of transcripts related to necroptosis, fibrosis, and inflammation demonstrated significantly increased expression in the kidneys of *Agxt*^−^/^−^ mice (Figure 4D–F). These results indicate that GA robustly induces nephrocalcinosis and renal injury in *Agxt*^−^/^−^ mice and is accompanied by activation of necroptotic, fibrotic, and inflammatory pathways, highlighting its superiority over EG-based induction methods in this model.

### 3.4. AAV843-hAAT-AGXT Gene Replacement Therapy Rescued the Phenotype of PH1 in Agxt^−^/^−^ Mice

**Figure 5:**
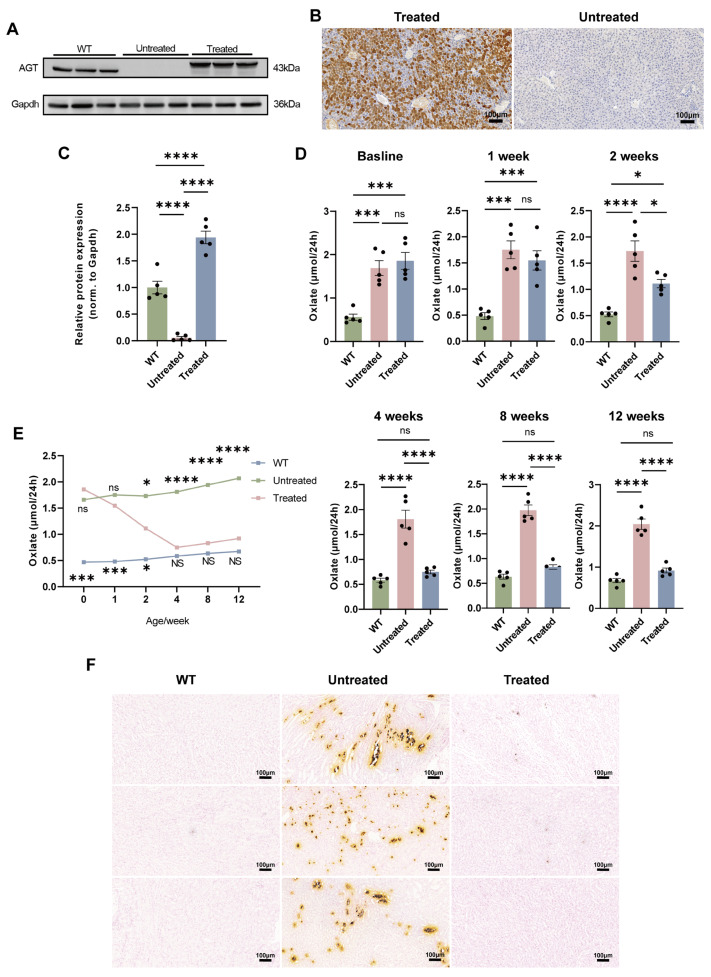
Restoration of hepatic AGT protein content and reduction in urinary oxalate levels after gene replacement therapy. (**A**,**C**) Western blotting and quantification of hepatic AGT protein contents in mice from each group following tail vein injection of AAV843-hAAT-AGXT for 12 weeks; Mann–Whitney U test. (**B**) Immunohistochemical staining of AGT in liver tissues from treated and untreated mice 12 weeks post-treatment. (**D**) Comparison of 24 h urinary oxalic acid contents in the WT, untreated, and treated groups at different time points during treatment. (**E**) Line graphs of 24 h urinary oxalic acid contents at different time points in each group. (**F**) Pizzolatto staining plots of kidney tissues of the WT, untreated, and treated groups after intraperitoneal injection of GA. Each group *n* = 5. Results are mean ± standard error. Scale bar = 100 μm. One-way analysis of variance (**D**,**E**), * *p* < 0.05, *** *p* < 0.001, **** *p* < 0.0001. ns, non-significant.

After validating the disease phenotype and optimizing the protocol for inducing renal calcium oxalate deposition, *Agxt*^−^/^−^ mice were injected via the tail vein with a single dose of AAV843-hAAT-AGXT to evaluate therapeutic efficacy. Western blotting and immunohistochemical analyses revealed robust re-expression of hepatic AGT protein in treated *Agxt*^−^/^−^ mice, whereas AGT remained undetectable in untreated controls (Figure 5A–C). Consistent with hepatic enzyme restoration, urinary oxalate excretion was significantly reduced in the treated group, falling below levels observed in untreated mice by week 2 and reaching values comparable to those of WT animals by week 4, where they remained stable thereafter (Figure 5D,E). Evaluation of renal calcium oxalate deposition by Pizzolato staining showed extensive crystal accumulation in untreated *Agxt*^−^/^−^ mice. At the same time, treated animals showed a significant reduction in renal calcium deposits, approaching the minimal levels observed in the WT controls (Figure 5F). These results demonstrate that AAV843-hAAT-AGXT gene delivery efficiently restores hepatic AGT expression, normalizes urinary oxalate levels, mitigates renal calcium deposition, and effectively rescues the disease phenotype in *Agxt*^−^/^−^ mice.

### 3.5. AAV843-hAAT-AGXT Gene Replacement Therapy Mitigated Kidney Injury in Agxt^−^/^−^ Mice

**Figure 6:**
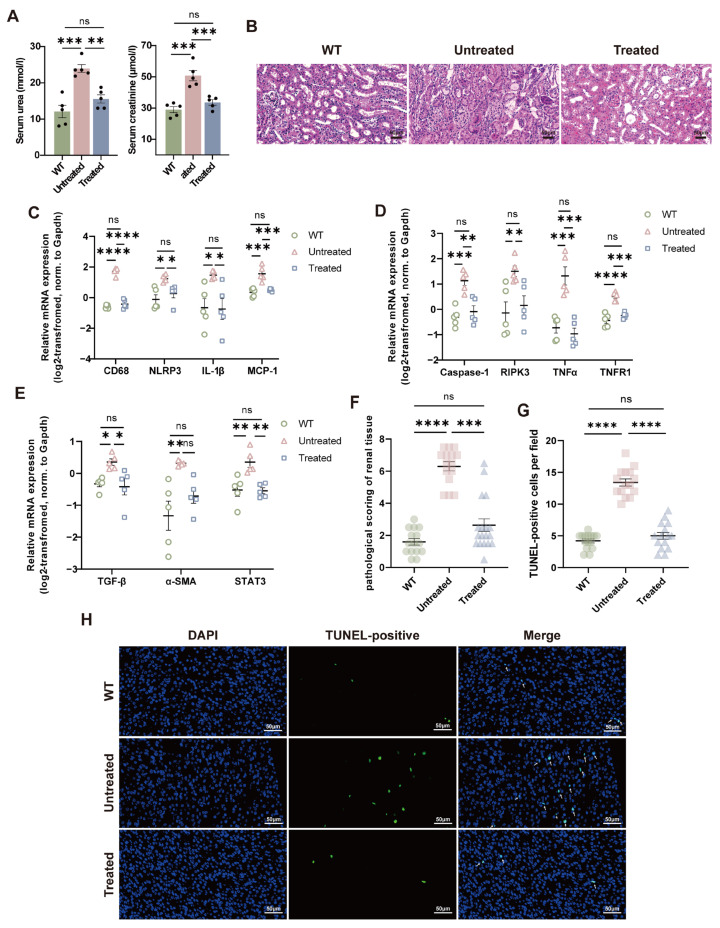
In vivo gene replacement therapy with AAV843-hAAT-AGXT reduces kidney injury in *Agxt*^−^/^−^ mice. (**A**) Blood urea and creatinine levels in the WT, untreated, and treated mouse groups after intraperitoneal injection of GA. (**B**) H&E staining of kidney sections from WT, untreated, and treated mice after GA-induced formation of renal stones; scale bar = 50 μm. (**C**) Expression of inflammation-associated factors in the renal tissues of the three groups. (**D**) Expression of necrosis-associated factors in the three groups. (**E**) Expression of fibrosis-associated factors in the three groups. (**F**,**G**) Semi-quantitative analysis of H&E staining and TUNEL staining, using three randomly selected fields per sample. (**H**) TUNEL staining of renal tissues from the three groups; scale bar = 50 μm. *n* = 5 per group. Data are presented as mean ± standard error. Significance was determined with (**A**,**C**–**E**) one-way analysis of variance and (**F**,**G**) a Kruskal–Wallis test. * *p* < 0.05, ** *p* < 0.01, *** *p* < 0.001, **** *p* < 0.0001. ns, non-significant.

Renal stone formation accompanied by progressive kidney injury and functional decline is a hallmark of PH1; therefore, we next evaluated the renoprotective effects of AAV843-hAAT-AGXT therapy. Serum urea and creatinine concentrations were significantly reduced in treated *Agxt*^−^/^−^ mice compared with untreated controls. They were restored to levels comparable to those observed in the WT animals (Figure 6A). Histopathological assessment revealed the most severe renal lesions in untreated mice, characterized by focal necrosis of glomerular vascular structures and extensive destruction or loss of tubular lumina. In comparison, renal damage was significantly attenuated in the treated group, while no pathological alterations were detected in WT kidneys (Figure 6B,F). Consistent with these findings, analysis of renal injury–associated markers demonstrated significantly lower expression of inflammatory (cluster of differentiation 68, CD68), necroptotic (receptor-interacting protein kinase 3, RIPK3), and fibrotic (alpha-smooth muscle actin, α-SMA) proteins in treated mice relative to untreated controls (Figure 6C–E). Furthermore, TUNEL staining revealed a pronounced reduction in renal apoptosis following treatment, with levels approaching those observed in WT mice (Figure 6G,H). These results indicate that AAV843-hAAT-AGXT gene replacement therapy effectively mitigates renal injury and suppresses inflammation, fibrosis, and apoptosis-related pathways in *Agxt*^−^/^−^ mice.

### 3.6. Evaluation of AAV843-hAAT-AGXT Safety

**Figure 7:**
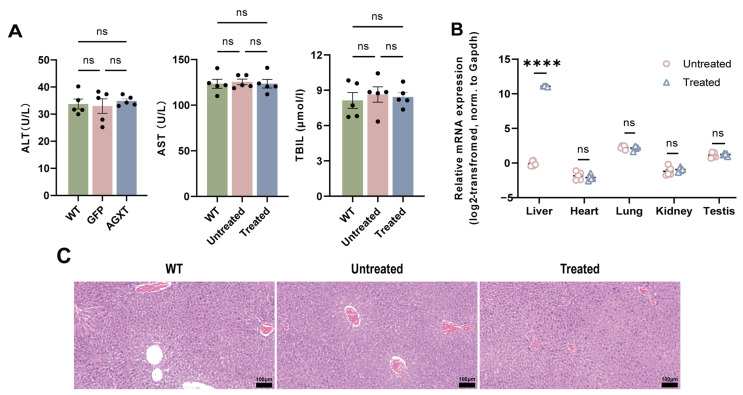
Evaluation of the safety of AAV843-hAAT-AGXT in vivo gene therapy. (**A**) Serum ALT, AST, and TBIL levels in WT, untreated, and treated mice at 12 weeks post-treatment, *n* = 5 per group. One-way analysis of variance. (**B**) mRNA expression levels of *AGXT* in various organs from untreated and treated mice using human *AGXT* primers; *n* = 5 per group. Unpaired Student’s *t*-test. (**C**) H&E staining of liver tissues from WT, untreated, and treated mice; *n* = 5 per group. Scale bar = 100 μm, **** *p* < 0.0001. ns, non-significant.

The safety of AAV843-hAAT-AGXT treatment was further assessed in vivo. Serum alanine aminotransferase (ALT), aspartate aminotransferase (AST), total bilirubin(TBIL) levels did not differ significantly among the experimental groups (Figure 7A). Histopathological analysis demonstrated preserved hepatic architecture without evidence of inflammatory cell infiltration (Figure 7C), indicating the absence of hepatotoxic effects. *AGXT* mRNA expression was primarily confined to the liver, with only negligible expression detected in extrahepatic tissues, confirming the high degree of liver specificity achieved by AAV843-hAAT-AGXT (Figure 7B). These results indicate that AAV843-hAAT-AGXT displays a favorable safety profile. All mice completed the full experimental protocol throughout the study without the need for premature euthanasia.

## 4. Discussion

In this study, we established the first GA-optimized protocol for inducing nephrocalcinosis in gene-edited PH1 mouse models. This approach reproducibly triggered renal calcium oxalate deposition exclusively in *Agxt*^−^/^−^ mice while sparing WT controls, providing a robust, genotype-dependent platform for therapeutic evaluation. In comparison, the EG–based induction method commonly used in previous studies proved unreliable in our model, displaying substantial inter-individual variability, prolonged induction periods, and minimal renal injury. These observations are consistent with earlier reports showing that only 28.5% of PH1 model mice exposed to 1.25% EG for four weeks developed mild-to-moderate calcium deposition [16]. The limited effectiveness of EG likely reflects the high environmental adaptability and strong glomerular filtration capacity of C57BL/6 mice, along with variability in EG absorption and metabolic conversion [16,25,32]. By comparison, intraperitoneal administration of GA, a direct metabolic precursor of oxalate, consistently induced significant nephrocalcinosis in all *Agxt*^−^/^−^ mice, with no detectable effects in WT littermates. GA bypasses the multistep metabolism required for EG and directly promotes oxalate accumulation, while also enhancing the expression of pro-calcific factors such as osteopontin (OPN) and bone morphogenetic protein 2 (BMP2), facilitating crystal aggregation [33,34,35]. Moreover, intraperitoneal delivery ensures rapid systemic exposure and more predictable pharmacokinetics, substantially improving experimental consistency and efficiency. Male mice showed significantly higher urinary oxalate levels than females, a finding that may be explained by testosterone-mediated enhancement of GO activity through androgen receptor-dependent mechanisms, leading to increased oxalate production and excretion [27,36,37].

Beyond model optimization, this study provides a comprehensive evaluation of renal injury following gene replacement therapy for PH1. Unlike previous reports that primarily focused on biochemical correction, we demonstrate that AAV843-hAAT-AGXT therapy not only reduces oxalate burden but also substantially attenuates renal injury by suppressing inflammatory, necroptotic, and fibrotic signaling pathways. Renal damage in PH1 is fundamentally driven by calcium oxalate (CaOx) crystal deposition, yet the resulting pathology arises from the coordinated activation of multiple interconnected signaling networks. CaOx crystals injure renal tubular epithelial cells, triggering endoplasmic reticulum stress and mitochondrial dysfunction, which in turn generate excessive reactive oxygen species (ROS). As a key upstream mediator, ROS has been reported to activate the NF-κB pathway and induce tubular cell apoptosis [38]. Similarly, NF-κB functions as a central inflammatory regulator, promoting the production of pro-inflammatory cytokines such as TNF-α and IL-1β [39]. These processes reinforce one another, amplifying inflammation, exposing the tubular basement membrane, and creating a microenvironment that favors crystal adhesion and plaque formation [39]. Consistent with this, our transcriptomic data revealed upregulation of NLRP3 pathway components. Previous studies have demonstrated that ROS can mediate NLRP3 inflammasome activation, suggesting the potential involvement of this pathway in the observed renal injury [40]. Transcriptomic analyses from this study further revealed aberrant activation of the PI3K–Akt signaling pathway. Dysregulation of this pathway may impair protective autophagy by activating mTOR and suppressing the transcription factor TFEB, aggravating inflammation and crystal retention [40]. At the same time, the concurrent upregulation of PI3K–Akt signaling and fibrotic markers such as TGF-β1 raises the possibility that this pathway may contribute to fibrosis, as has been suggested in previous studies [41]. Extensive crosstalk exists between these pathways: inflammatory mediators such as TNF-α can activate PI3K–Akt signaling, while Akt activation can further potentiate NF-κB activity, forming a self-amplifying loop that perpetuates inflammation and fibrosis [42,43,44,45]. It should be noted that while these associations are consistent with published literature, the present study did not directly measure ROS or perform pathway inhibition experiments. Within this complex pathological framework, AAV843-hAAT-AGXT therapy not only limits crystal accumulation but also confers multi-level renal protection, offering an integrated mechanistic rationale for its therapeutic benefit in PH1.

AAV843-hAAT-AGXT may offer translational promise, although further studies are needed to address key clinical considerations. The therapy achieved robust restoration of hepatic AGT expression, sustained normalization of urinary oxalate levels, and significant attenuation of renal injury in *Agxt*^−^/^−^ mice. The engineered AAV843 vector, which shows a substantially lower prevalence of neutralizing antibodies than the natural AAV8 serotype, has demonstrated favorable safety and efficacy profiles in clinical trials for hemophilia and other indications. In this study, AAV843-mediated delivery achieved highly liver-specific transgene expression, with predominant *AGXT* mRNA localization in hepatic tissue and minimal expression in extrahepatic organs. This targeting precision is particularly advantageous, as mature hepatocytes demonstrate low turnover rates, reducing dilution of transgene expression, while the liver’s extensive vascularization and sinusoidal structure facilitate efficient AAV uptake [46]. From a safety perspective, treated mice displayed normal serum ALT, AST, and TBIL levels, with no histological evidence of hepatic inflammation or toxicity. Moreover, no neoplastic lesions were observed throughout the study duration, further supporting the favorable safety profile of this approach [47,48].

Pre-existing NAbs remain a major barrier to the clinical application of AAV-based gene therapies, as they can compromise vector transduction and therapeutic efficacy. Seroprevalence studies indicate that NAb levels vary geographically, are generally higher in females and Asian populations, and increase with age [49]. While antibodies against natural AAV8 are relatively uncommon in East Asian populations, NAbs against AAV843 are reported to be nearly undetectable in the Chinese population based on previous seroprevalence studies [19]. However, anti-AAV843 antibody levels were not directly assessed in the present study, and their potential impact on transduction efficiency remains to be determined in future investigations. Current clinical protocols often exclude patients exceeding fixed NAb titer thresholds; however, evidence from a hemophilia B gene therapy trial demonstrated sustained Factor IX expression even in individuals with pre-existing NAb titers as high as 678 [17]. These findings suggest that rigid exclusion criteria may need to be reassessed based on vector serotype and disease context. For seropositive patients, adjunctive strategies such as immunoadsorption or treatment with an IgG-cleaving endopeptidase may further expand eligibility for AAV-based therapies [50,51]. Future studies should therefore focus on a comprehensive evaluation of AAV843 seroprevalence across diverse populations and on elucidating mechanisms by which pre-existing antibodies influence therapeutic outcomes.

Compared with alternative genetic approaches for PH1, gene replacement therapy offers distinct advantages. RNAi therapies targeting hydroxyacid oxidase 1 (HAO1) or lactate dehydrogenase A (LDHA) effectively reduce urinary oxalate levels but require lifelong repeated dosing due to RNA instability and incur substantial financial costs [3,8]. Clustered Regularly Interspaced Short Palindromic Repeats (CRISPR)–Cas9–based strategies can achieve permanent metabolic correction but raise concerns regarding off-target effects, chromosomal rearrangements, oncogenic risk, and widespread pre-existing immunity to Cas9 proteins [14,52,53,54,55]. Although base-editing technologies circumvent double-strand DNA breaks and enable precise correction of specific mutations, their applicability is limited to select point mutations and constrained by delivery challenges related to editor size [56]. Moreover, the long-term metabolic consequences of sustained inhibition of enzymes such as HAO1 or LDHA remain unclear. In comparison, AAV843-mediated *AGXT* gene replacement provides a mutation-independent strategy with favorable immunogenicity, precise tissue targeting, and broad clinical applicability.

Several limitations of this study should be acknowledged. The optimal timing of intervention remains uncertain, as delayed treatment may be less effective in patients with established renal damage. In comparison, early intervention could potentially shorten therapeutic durability due to hepatocyte turnover [57]. Given the close physiological similarities between humans and non-human primates, further validation in primate models is essential to assess long-term safety and efficacy comprehensively. Furthermore, the relatively small sample size in each experimental group (*n* = 5), although consistent with exploratory preclinical studies and ethical reduction principles, may limit statistical power and warrant cautious interpretation of the findings.

In conclusion, we demonstrate that deletion of *AGXT* exons 3–8 recapitulates key features of PH1 and that GA challenge induces robust nephrocalcinosis with greater efficiency than traditional EG-based methods. AAV843-mediated human *AGXT* gene replacement effectively normalizes oxalate metabolism, alleviates renal calcification and injury, and shows a favorable safety profile, thereby providing a foundation for further translational research. While the present findings are promising, the relatively small sample size warrants cautious interpretation. Future studies with extended observation periods in mouse models are needed to evaluate long-term efficacy and safety, determine the optimal therapeutic window, and validate immunogenicity, biodistribution, and safety in large animal models.

## Data Availability

Supporting data on reasonable request. Raw data have been deposited to National Center for Biotechnology Information (NCBI) under the BioProject number PRJNA1289679.

## Supplementary Materials

The following supporting information can be downloaded at: https://www.mdpi.com/article/10.3390/cells15070629/s1, Supplementary Methods; Supplementary Tables: Table S1: genotyping primers; Table S2: primers used in this study; Supplementary Figures: Figure S1: Hepatic vector genome copies.

## Abbreviations

The following abbreviations are used in this manuscript:

PH1	primary hyperoxaluria type I
*AGXT*	alanine-glyoxylate aminotransferase gene
AGT	alanine-glyoxylate aminotransferase
LDH	lactate dehydrogenase
GO	glycolate oxidase
NMPA	National Medical Products Administration
WT	wild-type
EG	ethylene glycol
GA	glyoxylic acid
qPCR	real-time fluorescence quantitative PCR
H&E staining	Hematoxylin-eosin staining
HAO1	hydroxyacid oxidase 1
LDHA	lactate dehydrogenase A
RNAi	RNA interference
NAbs	neutralizing antibodies
RNA-seq	RNA sequencing
OPN	osteopontin
BMP2	bone morphogenetic protein 2
CaOx	calcium oxalate
ALT	alanine aminotransferase
AST	aspartate aminotransferase
TBIL	total bilirubin
CD68	cluster of differentiation 68
RIPK3	receptor-interacting protein kinase 3
α-SMA	alpha-smooth muscle actin
NF-κB	nuclear factor kappa-B
TNF-α	tumor necrosis factor alpha
ECM	extracellular matrix
PI3K–Akt	phosphatidylinositol 3-kinase–protein kinase B
KEGG	Kyoto Encyclopedia of Genes and Genomes
TUNEL	Terminal deoxynucleotidyl transferase dUTP nick end labeling
ITR	inverted terminal repeats
hAAT	human α1 antitrypsin
UTR	5′ untranslated region
WPRE	woodchuck hepatitis virus post-transcriptional regulatory element
hGH-polyA	3′ human growth hormone polyadenylation signal
AAV	adeno-associated virus
CRISPR	Clustered Regularly Interspaced Short Palindromic Repeats
