# Supplementary material for: Novel AAV843 Vector-Mediated Gene Replacement Therapy Rescues Primary Hyperoxaluria Type I in Mice
Source: Cells. 2026 Mar 31;15(7):629. doi: 10.3390/cells15070629 (PMC13072227; doi:10.3390/cells15070629)
Supplement: Supplementary file 1 [file cells-15-00629-s001.zip › Supplementary Figures.pdf]

Supplementary Figures

Figure S1. Hepatic vector genome copies.

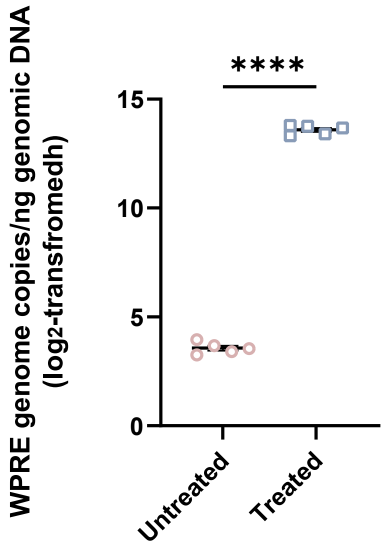

Quantification of hepatic WPRE genome copy numbers. Genome copy numbers were measured in the liver tissues of untreated and treated mice. Data are presented as mean ± SEM (n = 5 per group). Statistical significance was determined by an unpaired Student's t-test, ****$p < 0.0001$.
