# Supplementary material for: Novel AAV843 Vector-Mediated Gene Replacement Therapy Rescues Primary Hyperoxaluria Type I in Mice
Source: Cells. 2026 Mar 31;15(7):629. doi: 10.3390/cells15070629 (PMC13072227; doi:10.3390/cells15070629)

Figure1B.

AGT

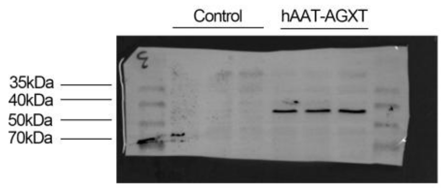

GAPDH

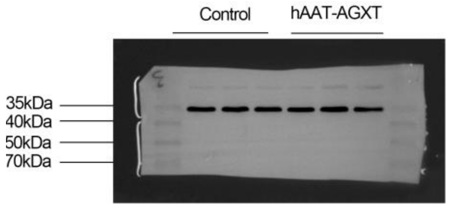

Repeated2:

AGT

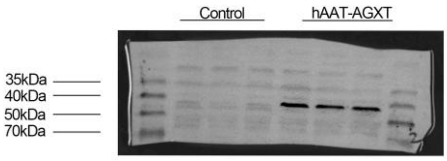

GAPDH

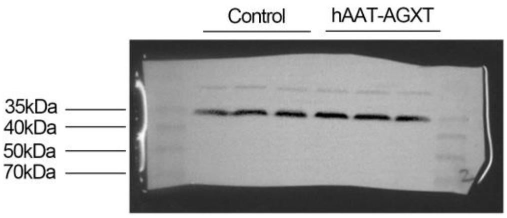

Repeated3:

AGT

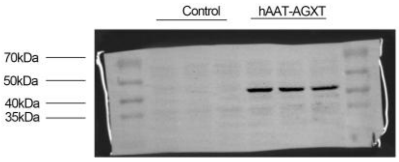

GAPDH

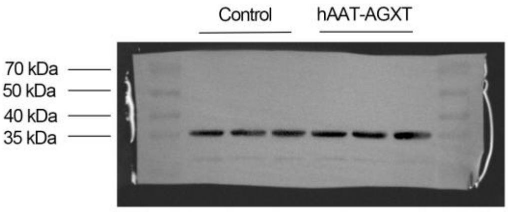

Figure2B.

AGT

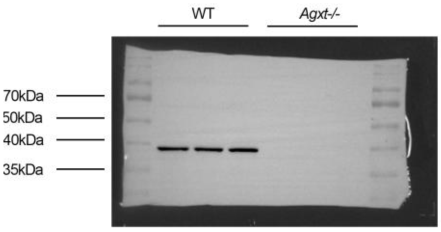

GAPDH

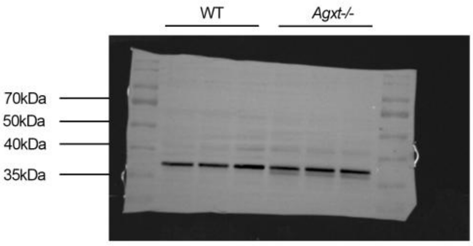

AGT

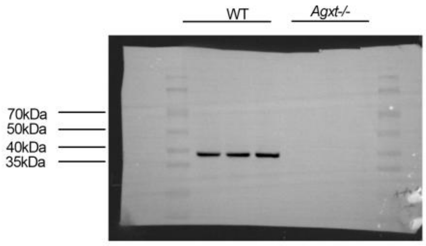

GAPDH

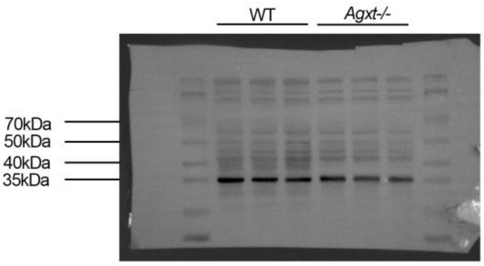

Repeated3:

AGT

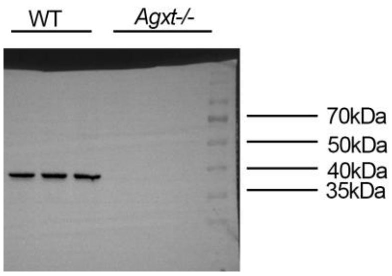

GAPDH

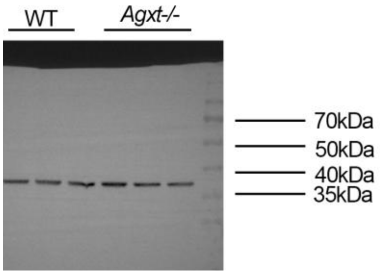

Figure5A

AGT

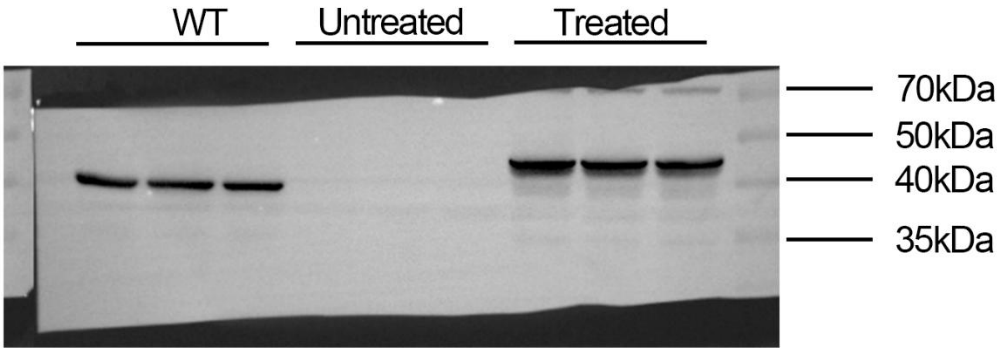

GAPDH

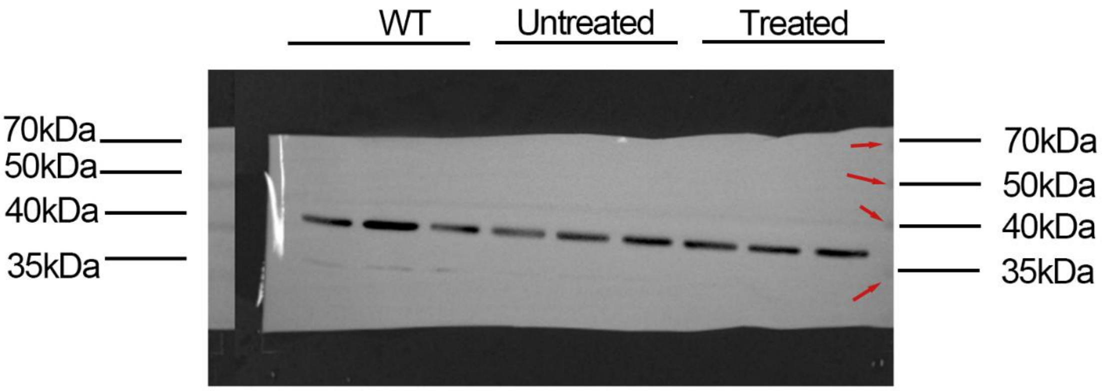

Repeated2:

AGT

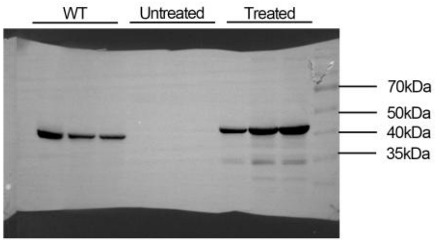

GAPDH

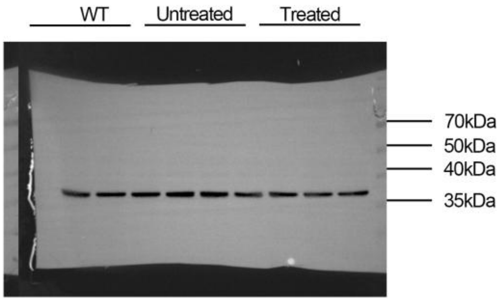

Repeated3:

AGT

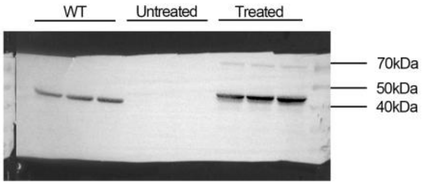

GAPDH

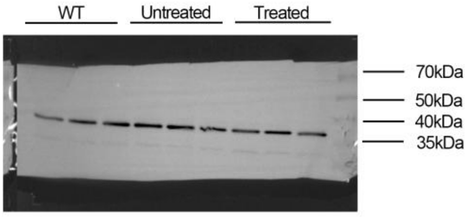

Supplement: Supplementary file 1 [file cells-15-00629-s001.zip › Supplementary Materials-Raw Data.pdf]
