# Supplementary material for: Novel AAV843 Vector-Mediated Gene Replacement Therapy Rescues Primary Hyperoxaluria Type I in Mice
Source: Cells. 2026 Mar 31;15(7):629. doi: 10.3390/cells15070629 (PMC13072227; doi:10.3390/cells15070629)
Supplement: Supplementary file 1 [file cells-15-00629-s001.zip › Supplementary Methods.pdf]

Supplementary Methods

Cell Culture and Transfection

Murine hepatoma Hepa1-6 cells were purchased from Shanghai Zhong Qiao Xin Zhou Biotechnology Co., Ltd. (China). The cells were cultured in DMEM (Gibco, 11995965) with 10% fetal bovine serum (Gibco, 10099141C) and 100 U/ml of penicillin and streptomycin (Gibco, 15140122). Transfection was performed in 6-well plates using Lipofectamine 3000 (Thermo Fisher, USA). At 70–80% confluence, the cell culture medium was replaced with Opti-MEM™ (Gibco, 31985070). For each well, the transfection mixture was prepared as follows: 2.5 μg of plasmid DNA was diluted in 125 μL Opti-MEM™, followed by the addition of 5 μL P3000™ reagent. Separately, 3.75 μL Lipofectamine™ 3000 was diluted in 125 μL Opti-MEM™ and incubated at room temperature for 5 min. The two solutions were then combined, incubated for 15 min at room temperature to form transfection complexes in a final volume of 261.25 μL, and added to the corresponding well. Cells in the experimental group (hAAT-AGXT) were transfected with the constructed plasmid containing the target sequence, while control wells received an equal volume of transfection mixture without plasmid. After 6 h, the medium was replaced with 2 mL of complete medium per well, and cells were continued in culture.

Blood and Urine Sample Processing

The blood samples were allowed to stand at room temperature for 1 h, followed by centrifugation at 2500 rpm for 10 min. The serum levels of creatinine, urea nitrogen, alanine aminotransferase (ALT), aspartate aminotransferase (AST), and total bilirubin were measured using a fully automated biochemical analyzer (Servicebio, China). Prior to urine collection, hydrochloric acid was added to the collection tubes to adjust the pH of the urine to below 2. The volume and weight of the urine were measured, and a 200 μL aliquot was diluted tenfold and filtered. Finally, oxalate ions were quantified using ion chromatography (Dionex ICS-5000, Thermo Fisher).

Pizzolatto staining

Deparaffinized sections were treated with an incubation solution, which was applied dropwise. The incubation solution was prepared by mixing calcium oxalate A solution (Servicebio, 10011218) and calcium oxalate B solution (Servicebio, G0150-500ML) in a 1:1 ratio. The sections were then exposed to UV light for 3 min, followed by staining with a nuclear solid red solution for 3-5 min. The sections were dehydrated and sealed prior to microscopic analysis.

TUNEL staining

Paraffin sections of kidney tissues were analyzed using a TUNEL kit (Servicebio; G1501), according to the provided instructions, to assess cell apoptosis.

Bulk RNA sequencing

Total RNA was isolated using TRIzol reagent (Invitrogen, USA; 15596018) following the manufacturer's instructions. Sequencing libraries were prepared with the VAHTS Universal V10 RNA-seq Library Prep Kit (Premixed Version) and subjected to paired-end sequencing on an Illumina NovaSeq 6000 platform. Raw sequencing reads in FASTQ format were quality-controlled and adapter-trimmed using fastp software (version 0.11.2). The cleaned reads were then aligned to

the reference genome using HISAT2 (version 2.1.0), and gene expression levels were quantified as FPKM values. Differential expression analysis was performed with the DESeq2 (version 1.22.2), applying thresholds of an adjusted Q-value < 0.05 (Benjamini-Hochberg) and an absolute fold change > 2 for defining significance. Finally, functional enrichnt analyses—specifically Gene Ontology (GO) and Kyoto Encyclopedia of Genes and Genomes (KEGG) analyses—were performed to identify differentially expressed genes.

Vector Genome Copy Number Analysis

Total DNA was extracted from liver tissues using the SteadyPure Universal Genomic DNA Extraction Kit (Accurate Biotechnology, AG21009) according to the manufacturer's instructions. WPRE genome copy numbers in the liver were quantified by real-time quantitative PCR (qPCR) using primer sets listed in Table S2. A standard curve for vector genome quantification was generated by serial dilution of the hAAT-AGXT plasmid containing the WPRE element. qPCR amplification was performed using a premix qPCR kit (Accurate Biotechnology; AG11718) on a Roche LightCycler 480II system, with thermal cycling conditions identical to those described in Section 2.5.
