# Supplementary material for: Novel AAV843 Vector-Mediated Gene Replacement Therapy Rescues Primary Hyperoxaluria Type I in Mice
Source: Cells. 2026 Mar 31;15(7):629. doi: 10.3390/cells15070629 (PMC13072227; doi:10.3390/cells15070629)
Supplement: Supplementary file 1 [file cells-15-00629-s001.zip › Supplementary Tables.pdf]

Supplementary Table.

Table S1. Genotyping primers.

Primer ID	Primer sequence	Primer ID	Primer sequence
Agxt-F1	TGAATAGGATGCTGCCTCTTCCAG	*Agxt*-R1	ACCCAGTTCTCATGCAGTGACTTC
Agxt-F2	CAGAGCCTTGGTTGTCTGTAAAATG	*Agxt*-R2	GAGGCTCTTGGCAGCCTTATCAC

Oligomers listed in 5'- to 3'- orientation.

Table S2. qRT-PCR primers.

Primer ID	Primer sequence	Primer ID	Primer sequence
Agxt-mouse-qPCR-F	CACGGGGAGTCATCCACTG	*Agxt*-mouse-qPCR-R	GAGTCCACCAGGAGTAGGCA
AGXT-human-qPCR-F	GACATCCTGTACTCGGGCTC	*AGXT*-human-qPCR-R	TGACGGGGATTGTGTGATGG
Gapdh-mouse-qPCR-F	TGTGTCCGTCGTGGATCTGA	*Gapdh*-mouse-qPCR-R	TTGCTGTTGAAGTCGCAGGAG
qPCR-CD68-F	CCAAGCCCAAATTCAAATCCG	qPCR-CD68-R	ATGGGTACCGTCACAACCT
qPCR-NLRP3-F	ATTGCTGTGTGTGGGACTGAA	qPCR-NLRP3-R	ATCCTGACAACACGCGGAT
qPCR-IL-β-F	TGGTGTGTGACGTTCCCATT	qPCR-IL-1β -R	TGTCGTTGCTTGGTTCTCCT
qPCR-MCP1-F	AGCAGCAGGTGTCCCAAAGA	qPCR-MCP1-R	GTGCTGAAGACCTTAGGGCAGA
qPCR-Caspase-1-F	TGCTTTCTGCTCTTCAACACCA	qPCR-Caspase-1-R	CCAAGTCACAAGACCAGGCATAT
qPCR-PIPK3-F	GTGCTACCTACACAGCTTGAAC	qPCR-PIPK3-R	CCCTCCCTGAAACGTGGAC
qPCR-TNFα-F	GCCAGGAGGGAGAACAGAAACTC	qPCR-TNFα-R	GGCCAGTGAGTGAAAGGGACA
qPCR-TNFR1-F	AACCAGTTCCAACGCTACCT	qPCR-TNFR1-R	GCACAACTTCATACACTCCTCATT
qPCR-TGF-β-F	ATACGCCTGAGTGGCTGTCT	qPCR- TGF-β-R	CGTGGAGTTTGTTATCTTTGCTGT
qPCR-α-SMA -F	CCCTGAAGAGCATCCGACAC	qPCR-α-SMA -R	CCAGAGTCCAGCACAATACCA
qPCR-mTOR-F	CTCCAGGGCATCAACGAGAG	qPCR- mTOR-R	CTCATCACGGGCTTGGTTCT
qPCR-STAT3-F	TCCTGGCACCTTGGATTGAG	qPCR- STAT3-R	TGTGCTGATAGAGGACATTGGA
Agxt-/--PCR1-F	TGAATAGGATGCTGCCTCTTCCAG	*Agxt-/-*-PCR1-R	ACCCAGTTCTCATGCAGTGACTTC
Agxt-/--PCR2-F	CAGAGCCTTGGTTGTCTGTAAAATG	*Agxt-/-*-PCR2-R	GAGGCTCTTGGCAGCCTTATCAC
qPCR-WPRE-F	CGCTATGTGGATACGCTGCTTT	qPCR-WPRE-F	AGAGACAGCAACCAGGATTTATACA

Oligomers listed in 5'- to 3'- orientation.
